# Supplementary material for: The Emerging Physiological Role of AGMO 10 Years after Its Gene Identification
Source: Life (Basel). 2021 Jan 26;11(2):88. doi: 10.3390/life11020088 (PMC7911779; doi:10.3390/life11020088)
Supplement: Supplementary file 1 [file life-11-00088-s001.pdf]

Supplementary Materials

Table S1. AGMO variants implicated in human diseases including the chromosomal position and the corresponding reference.

Variant	Mutation	Chromosomal Location (Start)	Mapped Gene(s)	Reference
rs2191349	G>C / G>T	7:15024684	AGMO, DGKB	[40,41,43]
rs10479944	T>A / T>G	7:15074211	AGMO, AC006458.1	[58]
rs12699683	G>T	7:15093502	AGMO, AC006458.1	[70]
rs115030349	T>C	7:15148284	AGMO, AC006458.1	[71]
	del ex2-8/13	7:15160118	AGMO	[74-75]
rs2908334	C>G / C>T	7:15166614	AGMO, AC006458.1	[55]
rs7783100	T>A / T>C / T>G	7:15187015	AGMO, AC006458.1	[65]
rs12531478	G>A	7:15200269	AGMO, AC006458.1	[54]
rs7777171	C>A / C>T	7:15201812	AGMO	[60]
rs6952254	T>A / T>C	7:15235692	AGMO	[68]
rs10232074	C>G	7:15260750	AGMO	[62]
rs12531027	T>C	7:15296311	AGMO	[51]
rs10279259	T>C	7:15314104	AGMO	[68]
rs9648203	G>A / G>C / G>T	7:15316654	AGMO	[68]
rs73064535	T>C	7:15339805	AGMO	[69]
rs12699708	G>A / G>T	7:15363341	AGMO	[68]
rs139309795	G>A	7:15365564	AGMO	[83]
rs60424486	dupTTTGA-GAGGTGCCT	7:15370687	AGMO	[61]
rs368430933	G>A / G>C	7:15385575	AGMO	[81]
rs143439626	T>C	7:15390881	AGMO	[83]
rs73284431	G>C	7:15394605	AGMO	[53]
rs6960653	T>C / T>G	7:15395008	AGMO	[68]
rs183966018	C>T	7:15418914	AGMO	[64]
rs11765688	A>G	7:15428574	AGMO	[68]
rs7781293	C>A / C>G / C>T	7:15451358	AGMO	[50]
rs4628172	T>G	7:15455525	AGMO	[50]
rs4721442	T>G	7:15466382	AGMO	[57]
rs73290427	G>C / G>T	7:15466485	AGMO	[62]
rs55905169	G>A / G>C	7:15466904	AGMO	[59]
rs7789097	C>A / C>T	7:15468511	AGMO	[63]
rs6461187	C>A / C>G	7:15481733	AGMO	[58]
rs202094268	A>G	7:15544884	AGMO	[81]
	del ex1-3/13	7:15549000	TMEM195/AGMO	[85]

rs916943	C>T	7:15554143	AGMO	[56]
	G>A<br>G>T	7:15560278 and<br>7:15560266	AGMO	[82]
	G>A<br>delTAT	7:15560292 and<br>7:15560264	AGMO	[82]
rs10239845	A>G	7:15566713	AGMO, MEOX2	[68]
rs6951635	T>C	7:15571071	AGMO, MEOX2	[72]
rs6951635	T>C	7:15571071	AGMO, MEOX2	[73]
rs11763353	A>G	7:15591246	AGMO, MEOX2	[67]
rs397948882	dupA	7:15597254	AGMO, MEOX2	[64]
rs1859270	T>A / T>C / T>G	7:15604673	AGMO, MEOX2	[52]
rs12699747	T>A / T>C / T>G	7:15610603	AGMO, MEOX2	[66]
